# Quantum mechanics of particles constrained to spiral curves with application to polyene chains

**DOI:** 10.1007/s00894-024-06030-y

**Published:** 2024-07-01

**Authors:** Eduardo V. S. Anjos, Antonio C. Pavão, Luiz C. B. da Silva, Cristiano C. Bastos

**Affiliations:** 1 Department of Fundamental Chemistry, Federal University of Pernambuco, Recife, 50740–540 Brazil; 2 Division of Mathematics, School of Science and Engineering, University of Dundee, Dundee, DD1 4HN UK; 3 Department of Chemistry, Rural Federal University of Pernambuco, Recife, 52171–900 Brazil

**Keywords:** Geometry-induced potential, Differential geometry, Bessel wave functions, Polyenes, $\pi$ electrons, Effective mass

## Abstract

**Context:**

Due to advances in synthesizing lower-dimensional materials, there is the challenge of finding the wave equation that effectively describes quantum particles moving on 1D and 2D domains. Jensen and Koppe and Da Costa independently introduced a confining potential formalism showing that the effective constrained dynamics is subjected to a scalar geometry-induced potential; for the confinement to a curve, the potential depends on the curve’s curvature function.

**Method:**

To characterize the $\varvec{\pi }$ electrons in polyenes, we follow two approaches. First, we utilize a weakened Coulomb potential associated with a spiral curve. The solution to the Schrödinger equation with Dirichlet boundary conditions yields Bessel functions, and the spectrum is obtained analytically. We employ the particle-in-a-box model in the second approach, incorporating effective mass corrections. The $\varvec{\pi }$-$\varvec{\pi ^{*}}$ transitions of polyenes were calculated in good experimental agreement with both approaches, although with different wave functions.

## Introduction

Thanks to tremendous advances in experimental techniques, synthesizing lower-dimensional materials became a reality (see Ref. [1] and references therein). Such materials often display formidable properties that offer countless opportunities. With such advances comes the challenge of finding the wave equation that effectively describes quantum particles moving on 1D and 2D materials. To find the effective wave equation for a particle confined to move on a lower-dimensional region, it is necessary to account for the uncertainty relations since any confinement involves the full knowledge of the degrees of freedom associated with the motion along the direction orthogonal to the constraining region. In the 1950s, De Witt attempted to describe quantum confinement in a curved space through a quantization procedure, which resulted in an ordering ambiguity [2]. A formalism that does not suffer from this ambiguity has been proposed independently by Jensen and Koppe [3] in the 1970s and by Da Costa in the 1980s [4]: their formalism shows that the effective constrained dynamics is subjected to a scalar geometry-induced potential. Jensen and Koppe analysed a case where confinement occurs between two parallel surfaces. They obtained that the Schrödinger equation depends on a geometry-induced potential $V_{gip}$ that incorporates the geometry of the confinement region [3]. On the other hand, Da Costa arrived at the same result by employing an explicit strong confining potential to restrict the particle’s motion to the desired lower-dimensional region [4]; for the confinement of a quantum particle to a curve, he obtained a Hamiltonian whose geometry-induced potential depends on the curve’s curvature function.

Several works have exploited the Jensen-Koppe-Da Costa’s formalism. For example, there are studies of charge transport in semiconductors or carbon nanostructures [5–7]. Del Campo et al. studied geometry-induced potentials that result in better transmittance in bent waveguides [5]. Da Silva et al. studied the problem of prescribed geometry-induced potential for invariant surfaces, showing that the probability density distribution can be controlled if we add an extra charge to the surface [6]. Lima et al. calculated the energy and analysed the implications of the geometry-induced potential for confinement in a helix, catenary, helicoid, and catenoid, concluding that for the helix, the angular momentum is quantized due to the geometry and that in the other cases, a continuous energy band of excited states appears [7]. Experimentally, Onoe et al. reported the observation of effects due to the geometry-induced potential on the Tomanaga-Luttinger liquid exponent in a 1D metallic $C_{60}$ polymer with an uneven periodic peanut-shaped structure [8]. As an alternative to Jensen-Koppe-Da Costa’s formalism, Bastos et al. studied the effects of intrinsic geometry on a particle confined in a generalized cylinder with a smooth cross-section. They noted that the topology, and not the geometry of the cross-section, plays a fundamental role in solving the problem in a Möbius strip and aromatic molecules [9].

Electrons moving in the ballistic regime are less affected by the lattice structure and can often be described as free particles if we properly renormalize their mass [10], thus giving rise to the concept of effective mass. A similar situation happens for $\pi$ electrons, i.e. electrons on a $\pi$ bond. The $\pi$ bonds are usually weaker than sigma bonds. Consequently, $\pi$ electrons can sometimes be reasonably described as a particle in a box [11–13]. The $\pi$ electron wave functions are the usual trigonometric functions in such a regime.

In this work, we provide an alternative description of $\pi$ electrons by modelling them confined to a spiral-like curved 1D box. By incorporating a spiral behaviour, the new wave functions are given by certain Bessel functions. Bessel functions exhibit a more complex behaviour than the usual trigonometric functions and introduce new factors, such as zero modes and wave amplitude dependence on the energy level. We apply this idea to characterize the $\pi$ electrons in linear polyenes chains (Fig. 2). Specifically, we solve the Schrödinger equation for a particle confined in certain spiral curves that can describe the 1D hydrogen atom and polyene linear chains. Our findings suggest a correlation between the electronic confinement, the effective mass, and the geometry-induced potential.

This work is divided as follows. The “Constrained quantum dynamics on plane curves” section presents Jensen-Koppe-Da Costa’s formalism for quantum particles constrained to move on a plane curve. The “1D hydrogen atom as a constrained quantum dynamics problem” section discusses the geometry of plane curves with power-law curvature functions and applies it to the 1D hydrogen atom seen as a constrained quantum dynamics problem. The “ Polyene chains as a constrained quantum dynamics problem” section introduces the family of plane curves that will be used to model $\pi$ electrons on polyene chains and presents the corresponding energy spectrum. Finally, in the “Conclusion” section, we present our concluding remarks.

## Constrained quantum dynamics on plane curves

**Fig. 1:**
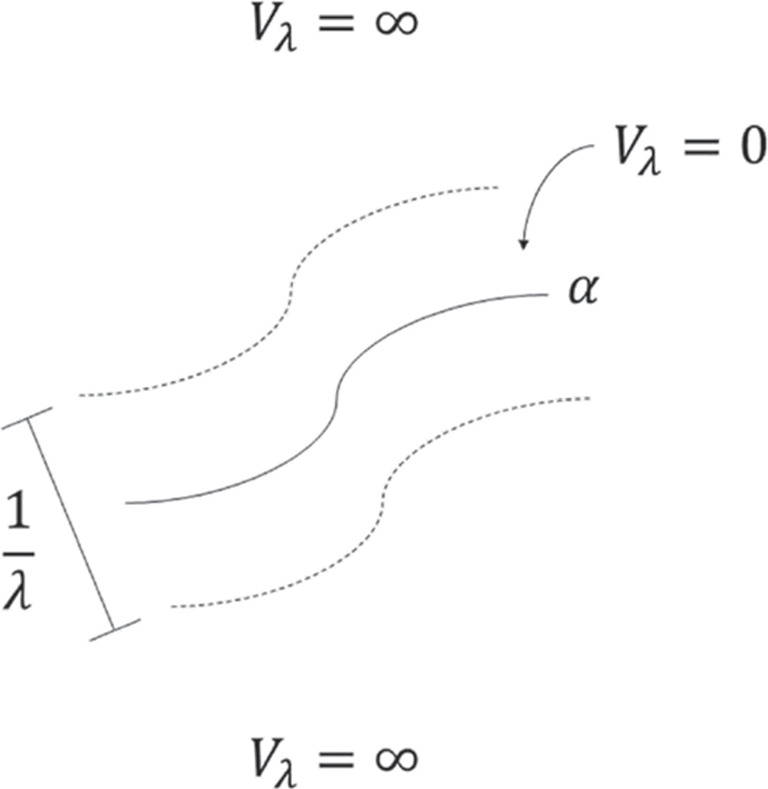
Particle-in-a-box constraining potential in the confinement of a quantum particle to a plane curve $\alpha :[a,b]\subseteq \mathbb {R}\rightarrow \mathbb {R}^2$. The particle is subject to a potential $V_{\lambda }$ that is zero on the points whose distance from $\alpha$ is smaller than or equal to $\frac{1}{2}\lambda ^{-1}$, and infinity if otherwise. In the limit $\lambda \rightarrow \infty$, one obtains the behaviour (1)

Assume we want to describe the motion of a quantum particle of mass *m* constrained to move along a plane curve $\alpha :[a,b]\rightarrow \mathbb {R}^2$. To find the equations for the constrained dynamics, we could follow Jensen and Koppe [3] and describe the confinement by starting from the dynamics in the region between two neighbouring parallel curves and imposing homogeneous boundary conditions along them. If we denote the distance between the two neighbouring curves by $1/\lambda$, then taking the limit $\lambda \rightarrow \infty$, one obtains the equations that govern the constrained dynamics. In other words, Jensen and Koppe considered the confinement via a particle-in-a-box model: the particle is subject to a potential $V_{\lambda }$ such that $V_{\lambda }(\vec {r})=0$ if the distance from $\vec {r}\in \mathbb {R}^2$ to $\alpha$ is smaller than or equal to $\frac{1}{2}\lambda ^{-1}$, and $V_{\lambda }(\vec {r})=\infty$ if otherwise (see Fig. 1).

Alternatively, following Da Costa [4], we may apply a family of explicit strong confining potentials $V_{\lambda }$ to restrict the particle’s motion to the desired curve:

1 $$\begin{aligned} \lim _{\lambda \rightarrow \infty } V_{\lambda }(\vec {r}) = \left\{ \begin{array}{ccc} 0 &{} , &{} \vec {r} \in \alpha \\[4pt] \infty &{} , &{} \vec {r} \not \in \alpha \\ \end{array} \right. . \end{aligned}$$

These procedures allow us to decouple the tangential and normal degrees of freedom in the limit $\lambda \rightarrow \infty$. In other words, one separates the Hamiltonian into a term that governs the low energy motion in the tangent direction, which is the effective Hamiltonian along the constraint region, and a high energy motion in the normal direction.

Employing the Jensen-Koppe-Da Costa formalism is necessary to account for the uncertainty relations since any confinement involves the full knowledge of some degrees of freedom, namely the motion along the directions orthogonal to the constraining region. De Witt’s description of quantum confinement in a curved space resulted in an ordering ambiguity [2]. The Jensen-Koppe-Da Costa formalism does not suffer from this ambiguity. In addition, it shows that the effective constrained dynamics is subjected to a scalar geometry-induced potential $V_{gip}$.

The (effective) Schrödinger equation for a quantum particle constrained to move on a plane curve $\alpha (s) = (x(s), y(s))$ is given by [4]

2 $$\begin{aligned} -\frac{\hbar ^2}{2m}\frac{d^2\psi (s)}{ds^2} +V_{gip}\,\psi (s) = E\psi (s), \quad V_{gip} = -\frac{\hbar ^2}{8m}k(s)^2, \end{aligned}$$

where *s* denotes the arc length parameter, i.e. $\alpha '(s)\cdot \alpha '(s)=1$, and *k* is the curvature function; $k(s)=\Vert \alpha ''(s)\Vert$.

If we were to describe the constrained quantum dynamics on a plane curve $\alpha$ without the geometry-induced potential, then the resulting energy spectrum would not depend on the geometry of $\alpha$ but only on whether $\alpha$ is a closed or an open curve [14]. Indeed, solving Eq. (2) without $V_{gip}$ with homogeneous boundary conditions (open curve of length *L*) gives

3 $$\begin{aligned} E_{n}^{\text {op}} = \frac{h^2n^2}{8mL^2}, \end{aligned}$$

while solving (2) without $V_{gip}$ with periodic boundary conditions (closed curve of length *L*) gives

4 $$\begin{aligned} E_{n}^{\text {cl}} = 4E_{n}^{\text {op}} = \frac{h^2n^2}{2mL^2}. \end{aligned}$$

## 1D hydrogen atom as a constrained quantum dynamics problem

If we consider a function of the form $k(s) =\frac{1}{\sigma \sqrt{s}}$, where $\sigma$ is a real parameter, then confining a particle to move on a plane curve with curvature *k* can lead to a geometry-induced corresponding to the 1D hydrogen atom:

5 $$\begin{aligned} V_{gip} = -\frac{\hbar ^2}{8m}k(s)^2 = -\frac{\hbar ^2}{8m\sigma ^2s} = -K\frac{q_1q_2}{s}, \end{aligned}$$

where *K* denotes the permittivity of free space, $K=9\times 10^9 Nm^2/C$, $q_1$ denotes the charge of the nucleus, and $q_2$ the charge of the electron. Indeed, take the constant $\sigma = \sqrt{\frac{\hbar }{8mKq_1q_2}}$. We shall refer to a curve $\alpha _H:[a,b]\rightarrow \mathbb {R}^2$ whose corresponding curvature function *k* satisfies (5) as a *hydrogen curve*.

### Plane curves with power-law curvature function

A plane curve $\alpha (s)$ with curvature function *k*(*s*) can be parametrized as [15]

6 $$\begin{aligned} s \mapsto \alpha (s)=\left( \int _{s_0}^s \cos (\int _{s_0}^v k(u) \, du) \, dv,\int _{s_0}^s \sin (\int _{s_0}^v k(u) \, du) \, dv\right) . \end{aligned}$$

For a generic parametrization $\alpha (t)$, the arc length parameter can be obtained as a function of *t* by the expression $s=\int _{t_0}^t\Vert \dot{\alpha }(\tau )\Vert \,d\tau$, while the curvature function is $k=\Vert \dot{\alpha }\times \ddot{\alpha }\Vert /\Vert \dot{\alpha }\Vert ^3$.

The hydrogen curve belongs to the family of curves with a power-law curvature function

7 $$\begin{aligned} k(s) = \frac{1}{\sigma s^p}, \quad \sigma >0 \quad \text{ and } \quad p\in \mathbb {R}. \end{aligned}$$

The hydrogen curve $\alpha _H$ has $p=1/2$. Power-law curvature functions lead to spiral-like curves.

Every plane curve satisfies the Frenet equations

8 $$\begin{aligned} \left\{ \begin{array}{ccc} \textbf{t}'(s) &{} = &{} k(s)\,\textbf{n}(s)\\ \textbf{n}'(s) &{} = &{} -k(s)\,\textbf{t}(s)\\ \end{array} \right. \,, \end{aligned}$$

where $\textbf{t}=\alpha '$ denotes the curve’s unit tangent and $\textbf{n}$ is the principal normal vector field. The geometric interpretation of the vector fields $\textbf{t}$ and $\textbf{n}$ is as follows. If we think of a plane curve as describing the motion of a particle in the plane, the unit normal $\textbf{n}$ points in the direction of the centripetal acceleration vector. Indeed, applying the chain rule, $\frac{d\alpha }{dt}=v\frac{d\alpha }{dt}$, $v=\Vert d\alpha /dt\Vert$, from which we obtain that $\frac{d^2\alpha }{dt^2} = \frac{dv}{dt}\textbf{t} + v^2k\,\textbf{n}$.

The solutions of Eq. 8 for the power-law case, Eq. 7, are given by

$$\begin{aligned} \textbf{t}(s)&= \textbf{a}\, C_p(s) + \textbf{b}\, S_p(s)\quad \text{ and } \quad \textbf{n}(s) \\&= \sigma s^p\,\textbf{t}'(s) = -\textbf{a}\,S_p(s)+\textbf{b}\,C_p(s), \end{aligned}$$

where $\textbf{a}$ and $\textbf{b}$ are constant vectors and the real functions $C_p$ and $S_p$ are defined as

9 $$\begin{aligned}&C_p(s) = \left\{ \begin{array}{ccc} \displaystyle \cos \left( \frac{s^{1-p}}{\sigma (1-p)}\right) &{} , &{} p \not = 1\\[9pt] \cos \left( \sigma ^{-1}\ln s\right) &{} , &{} p = 1\\ \end{array} \right. \quad \text {and} \quad \nonumber \\&S_p(s) = \left\{ \begin{array}{ccc} \displaystyle \sin \left( \frac{s^{1-p}}{\sigma (1-p)}\right) &{} , &{} p \not = 1\\[9pt] \sin \left( \sigma ^{-1}\ln s\right) &{} , &{} p = 1\\ \end{array} \right. . \end{aligned}$$

If $$p=\frac{1}{2}$$, then

10 $$\begin{aligned} \int C_{\frac{1}{2}}(s)\,ds = \sigma \sqrt{s}\,S_{\frac{1}{2}}(s)+\frac{\sigma ^2}{2}C_{\frac{1}{2}}(s)+c_1 \end{aligned}$$

and

11 $$\begin{aligned} \int S_{\frac{1}{2}}(s)\,ds = -\sigma \sqrt{s}\,C_{\frac{1}{2}}(s)+\frac{\sigma ^2}{2}S_{\frac{1}{2}}(s)+c_2\,, \end{aligned}$$

where $c_1$ and $c_2$ are arbitrary constants.

Assuming for simplicity that $\textbf{t}(s_0)=(1,0)$ and $\textbf{n}(s_0)=(0,1)$, integration of the unit tangent, $\alpha _H=\int ^s\textbf{t}$, allows us to explicitly parametrize the hydrogen curve as

12 $$\begin{aligned} \alpha _H(s)=R_{\frac{1}{2}}(s_0)\left( \begin{array}{cc} \frac{\sigma ^2}{2} &{} \sigma \sqrt{s}\\[5pt] -\sigma \sqrt{s} &{} \frac{\sigma ^2}{2}\\ \end{array} \right) \left( \begin{array}{c} C_{\frac{1}{2}}(s)\\[5pt] S_{\frac{1}{2}}(s)\\ \end{array} \right) +\alpha _0\,, \end{aligned}$$

where $\alpha _0\in \mathbb {R}^2$ is a constant point and we have defined a “rotation” matrix $R_p(s)$

13 $$\begin{aligned} R_p(s) = \left( \begin{array}{cc} C_p(s) &{} S_p(s)\\[5pt] -S_p(s) &{} C_p(s)\\ \end{array} \right) . \end{aligned}$$

Note that $\Vert \alpha _H(s)-\alpha _0\Vert =\sigma \sqrt{s+\frac{\sigma ^2}{4}} \sim s^{1/2}$, which shows that $\alpha _H$ spirals around a point.

### Solution of the 1D hydrogen atom

In the 1950s, Loudon solved the 1D hydrogen atom on the line [16]:

14 $$\begin{aligned} -\frac{\hbar ^2}{2m}\frac{d\psi ^2}{dx^2}-\frac{e^2}{\vert x\vert }\psi = E\psi , \end{aligned}$$

where *e* is the electric charge of the electron and $\psi$ is a complex function defined over the real line: $\psi :\mathbb {R}\rightarrow \mathbb {C}$. The difficulty of solving the 1D hydrogen atom lies in the existence of a pole at $x=0$. The idea is to solve the equation for the regions $x<0$ and $x>0$ and then join the two solutions at $x=0$ by approaching the actual potential as the limit of a nonsingular potential *V*(*x*), see, e.g., Fig. 1 of Ref. [16].

If we write the eigenfunction along the hydrogen curve as a function of the arc length parameter $s>0$, we have the following wave function along the curve

15 $$\begin{aligned} \psi = B\textrm{e}^{-\frac{z}{2}}\,z\,L_{N}^1(z),\quad z=\frac{2s}{Na_0}, \end{aligned}$$

where *B* is a normalizing constant, $a_0=\hbar ^2/me^2$, and $L_a^b(z)$ denotes an associated Laguerre polynomial. Note that this solution is not equal to the radial solution of the 3D hydrogen atom:

16 $$\begin{aligned} R_{N\ell }(r) = B_{N\ell },\textrm{e}^{-\frac{z}{2}}z^{\ell }L_{N}^{2\ell +1}(z),\quad z=\frac{2r}{Na_0}, \end{aligned}$$

where $B_{N\ell }$ is a normalizing constant. However, taking into account the use of spherical coordinates to describe the radial part, one obtains the same probability density in both cases: $dP_{1D}=\vert \psi _{1D}\vert ^2ds=dP_{3D}=r^2\vert \psi _{1D}\vert ^2dr$, where one must take $\ell =0$ in the 3D solution to compare the solutions in both dimensions properly. As expected, this means that in the 1D solution, only *s* orbitals make sense and, therefore, a 1D periodic table will have 2 columns only [17, 18].

## Polyene chains as a constrained quantum dynamics problem

The consideration of $\pi$ electrons is essential for the stability of certain carbon compounds, such as polyenes [19, 20]. In these compounds, $\pi$ electrons can often be approximated as particles in a box [11–13]. For a particle confined in a one-dimensional box of length *L*, the solution to the Schrödinger equation gives the wave function

17 $$\begin{aligned} \psi _n(s) = \sqrt{\frac{2}{L}} \sin \left( \frac{n\pi s}{L}\right) . \end{aligned}$$

The allowed energy levels, $E_n$, of the particle are quantized and given by

18 $$\begin{aligned} E_n = \frac{n^2\pi ^2\hbar ^2}{2mL^2}. \end{aligned}$$

This model provides a good approximation for conjugated molecules with minimal alternation of bond lengths. However, for systems with significant bond length alternation, such as long-chain polyenes, the model cannot adequately describe the finite absorption wavelength limit of the system, thus requiring adjustments [21].

It is possible to improve the particle-in-a-box model by incorporating an effective mass into the Laplacian operator. This approach involves assessing which mass value accurately predicts the wavelengths of experimental transitions. Specifically, the calculated effective masses were $0.531m_e$, $0.457m_e$, $0.440m_e$, and $0.384m_e$ for deca-2,4,6,8-tetraene, dodeca-2,4,6,8,10-pentaene, tetradeca-2,4,6,8,10,12-hexaene, and hexadeca-2,4,6,8,10,12,14-heptaene molecules, respectively. These values, inversely proportional to the increase in the chains’ length, align with expectations from models of ballistic electrons in nanostructures and crystals, which tend towards 0.173$m_e$ [22].

The $\pi$ electrons are not strongly bound to the chain, with interactions weaker than those between charges, especially noticeable in long-chain systems such as polyenes. In this work, we propose to describe $\pi$ electrons using confinement with geometry-induced potential

19 $$\begin{aligned} V_{\text {gip}} = - \frac{\hbar ^2}{8m\sigma ^2 s^2}. \end{aligned}$$

We aim to represent a kind of average interaction between the molecule and the $\pi$ electrons. However, this interaction would be electromagnetic, weaker than a conventional Coulomb interaction but stronger than dipole interactions or Van der Waals forces.

### Polyene curve

Let us denote by $\alpha _P$ a plane curve whose corresponding curvature function is of the form $k=\frac{1}{\sigma s}$, i.e. Equation 7 with $p=1$. We shall refer to $\alpha _P$ as a *polyene curve*.

For $p=1$, the auxiliary functions $C_p$ and $S_p$ defined in Eq. 9 have the form

20 $$\begin{aligned} \int C_{1}(s)\,ds = \frac{\sigma \,s}{1+\sigma ^2}\left[ S_1(s)+\sigma C_1(s)\right] + c_1 \end{aligned}$$

and

21 $$\begin{aligned} \int S_{1}(s)\,ds = -\frac{\sigma \,s}{1+\sigma ^2}\left[ C_1(s)-\sigma S_1(s)\right] + c_2\,, \end{aligned}$$

where $c_1$ and $c_2$ are arbitrary real constants.

Assuming for simplicity that $\textbf{t}(s_0)=(1,0)$ and $\textbf{n}(s_0)=(0,1)$, integration of the unit tangent, $\alpha _P=\int ^s\textbf{t}$, allows us to explicitly parametrize the polyene curve as

22 $$\begin{aligned} \alpha _P(s)=\frac{\sigma s}{1+\sigma ^2}\,R_{1}(s_0)\left( \begin{array}{cr} \sigma &{} 1 \\ 1 &{} -\sigma \\ \end{array} \right) \left( \begin{array}{c} C_{1}(s)\\ S_{1}(s)\\ \end{array} \right) +\alpha _0\,, \end{aligned}$$

where $\alpha _0\in \mathbb {R}^2$ is constant and $R_1(s)$ is defined as in Eq. 13.

Note that $\Vert \alpha _P(s)-\alpha _0\Vert =\frac{\sigma }{\sqrt{1+\sigma ^2}} s$, which shows that $\alpha _P$ spirals around a point, as happens for the hydrogen curve. Note that polyene curves approach its initial point $\alpha _0$ faster than the hydrogen curve: $\frac{\Vert \alpha _P(s)-\alpha _0\Vert }{\Vert \alpha _H(s)-\alpha _0\Vert } \overset{s\rightarrow 0}{\longrightarrow }0$. In addition, polyene curves rotate around its initial point $\alpha _0$ more than the hydrogen curve, as a comparison between $\{C_{1}(s),S_{1}(s)\}$ and $\{C_{\frac{1}{2}}(s),S_{\frac{1}{2}}(s)\}$ indicates.

### Spectrum of $\pi$ electrons in polyene chains

**Fig. 2:**
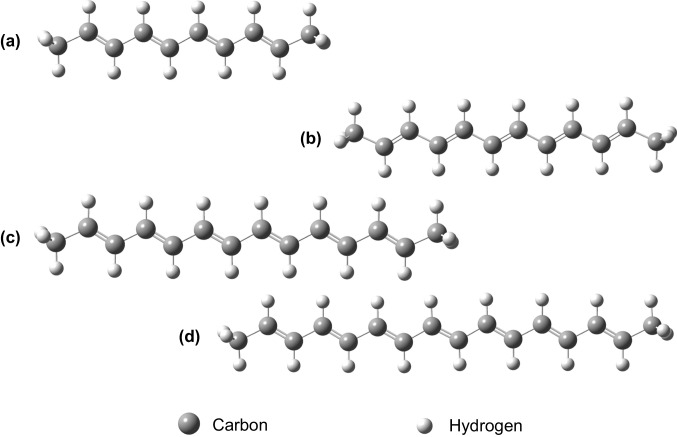
Polyenes. (a) deca-2,4,6,8-tetraene; (b) dodeca-2,4,6,8,10-pentaene; (c) tetradeca-2,4,6,8,10,12-hexaene; (d) hexadeca-2,4,6,8,10,12,14-heptaene

In this section, we provide an alternative description of $\pi$ electrons confined in a 1D curved box, as defined by the polyene curves discussed in the previous section. Bessel functions exhibit more complex behaviour than usual trigonometric functions and introduce new factors such as zero modes [23] and the dependence of wave amplitude on the energy level. We apply (2) to describe the $\pi$ electrons in the polyenes deca-2,4,6,8-tetraene, dodeca-2,4,6,8,10-pentaene, tetradeca-2,4,6,8,10,12-hexaene, and hexadeca-2,4,6,8,10,12,14-heptaene [24] (Fig. 2).

The polyene curve parametrization is given by

23 $$\begin{aligned} \alpha _P(s)= & {} \frac{\sigma s}{1+\sigma ^2}\Big (\cos \left( \frac{\ln s}{\sigma }\right) + \sigma \sin \left( \frac{\ln s}{\sigma }\right) ,\sin \left( \frac{\ln s}{\sigma }\right) \nonumber \\{} & {} - \sigma \cos \left( \frac{\ln s}{\sigma }\right) \Big ), \end{aligned}$$

where we have set $s_0=1$ and $\alpha _0=(0,0)$ in Eq. 22.

For the geometry-induced potential of polyenes curves, $V_{gip}=-\frac{\hbar ^2}{8m\sigma ^2 s^2}$, Eq. 2 becomes

24 $$\begin{aligned} -\frac{d^2\psi }{ds^2} = \left( \epsilon + \frac{1}{4\sigma ^2s^2}\right) \psi , \quad \epsilon = \frac{2mE}{\hbar ^2}, \end{aligned}$$

whose general solution is expressed as a linear combination of Bessel functions $J_{\omega }(s)$ and $Y_{\omega }(s)$ of the first and second types, respectively:

25 $$\begin{aligned} \psi _n (s)= & {} c_1 \sqrt{s}\,J_{\omega }(\sqrt{\epsilon }\,s) + c_2 \sqrt{s}\,Y_{\omega }(\sqrt{\epsilon }\,s), \nonumber \\ \omega= & {} \frac{1}{2}\sqrt{\left| 1-\frac{1}{\sigma ^2}\right| }\,. \end{aligned}$$

We want a solution on the interval [0, *L*] and, therefore, must impose the condition $c_2=0$ (Bessel functions of the second type diverge at the origin). Applying the homogeneous boundary conditions in Eq. 25, the solutions are subject to the relationship

26 $$\begin{aligned} L = \frac{j_{\omega , n}}{\sqrt{\epsilon }} \quad \Rightarrow \quad E_n = \frac{\hbar ^2}{2mL^2}\,j_{\omega , n}^2\,, \end{aligned}$$

where $j_{\omega , n}$ denotes the *n*-th zero of $J_{\omega }$.

To determine the value of $c_1$, we can use the normalization condition

27 $$\begin{aligned} 1 = \int _{0}^{L} |\psi _n (s)|^2 \,ds = c_1^2\int _{0}^{L} |\sqrt{s}\,J_{\omega }(\sqrt{\epsilon }\,s)|^2 \,ds, \end{aligned}$$

and obtain

28 $$\begin{aligned} c_1 = \frac{\sqrt{2}}{L\sqrt{J_{\omega }(\sqrt{\epsilon }\,s) - J_{\omega -1}(\sqrt{\epsilon }\,s)J_{\omega +1}(\sqrt{\epsilon }\,s)]}}. \end{aligned}$$

Using that $\epsilon = \frac{j_{\omega , n}}{L}$ and $J_{\omega }(j_{\omega , n}) = 0$, we have

29 $$\begin{aligned} c_1 = \frac{\sqrt{2}}{L\sqrt{- J_{\omega -1}(j_{\omega , n})J_{\omega +1}(j_{\omega , n})}}. \end{aligned}$$

Thus, the complete basis of wave functions is

30 $$\begin{aligned} \psi _n (s) = \frac{\sqrt{2}}{L\sqrt{-J_{\omega -1}(j_{\omega , n})J_{\omega +1}(j_{\omega , n})}}\,\sqrt{s}\,J_{\omega }\left( \frac{j_{\omega , n}}{L}s\right) . \end{aligned}$$

**Table 1:** Values of $\sigma$ and $\omega$ for distinct polyene chains

System	$\sigma$	$\omega$
deca-2,4,6,8-tetraene	$\sqrt{0.004}$	7.88987
dodeca-2,4,6,8,10-pentaene	$\sqrt{0.0014}$	13.35370
tetradeca-2,4,6,8,10,12-hexaene	$\sqrt{0.0009}$	16.65920
hexadeca-2,4,6,8,10,12,14-heptaene	$\sqrt{0.00045}$	23.56490

For each polyene, specific values of $\sigma$ and $\omega$ were obtained, representing system structure changes. Table 1 shows these values for four distinct systems. The value of $\sigma$ was determined using the relationship between the wavelength of transition $\lambda$ and the energy change $\Delta E$: $\lambda = \frac{hc}{\Delta E}$, where *c* is the velocity of light in vacuum and *h* the Planck constant. Comparison with experimental values of $\lambda$ [24] allows us to estimate the corresponding values of $\sigma$. Note that the appropriate energy levels used to compute $\Delta E$ depend on how many $\pi$ electrons the polyene chains have, namely, 8 for the deca-2,4,6,8-tetraene molecule ($n = 4$), 10 for the dodeca-2,4,6,8,10-pentaene molecule ($n = 5$), 12 for the tetradeca-2,4,6,8,10,12-hexaene molecule ($n = 6$), and 14 for the hexadeca-2,4,6,8,10,12,14-heptaene molecule ($n = 7$).

**Fig. 3:**
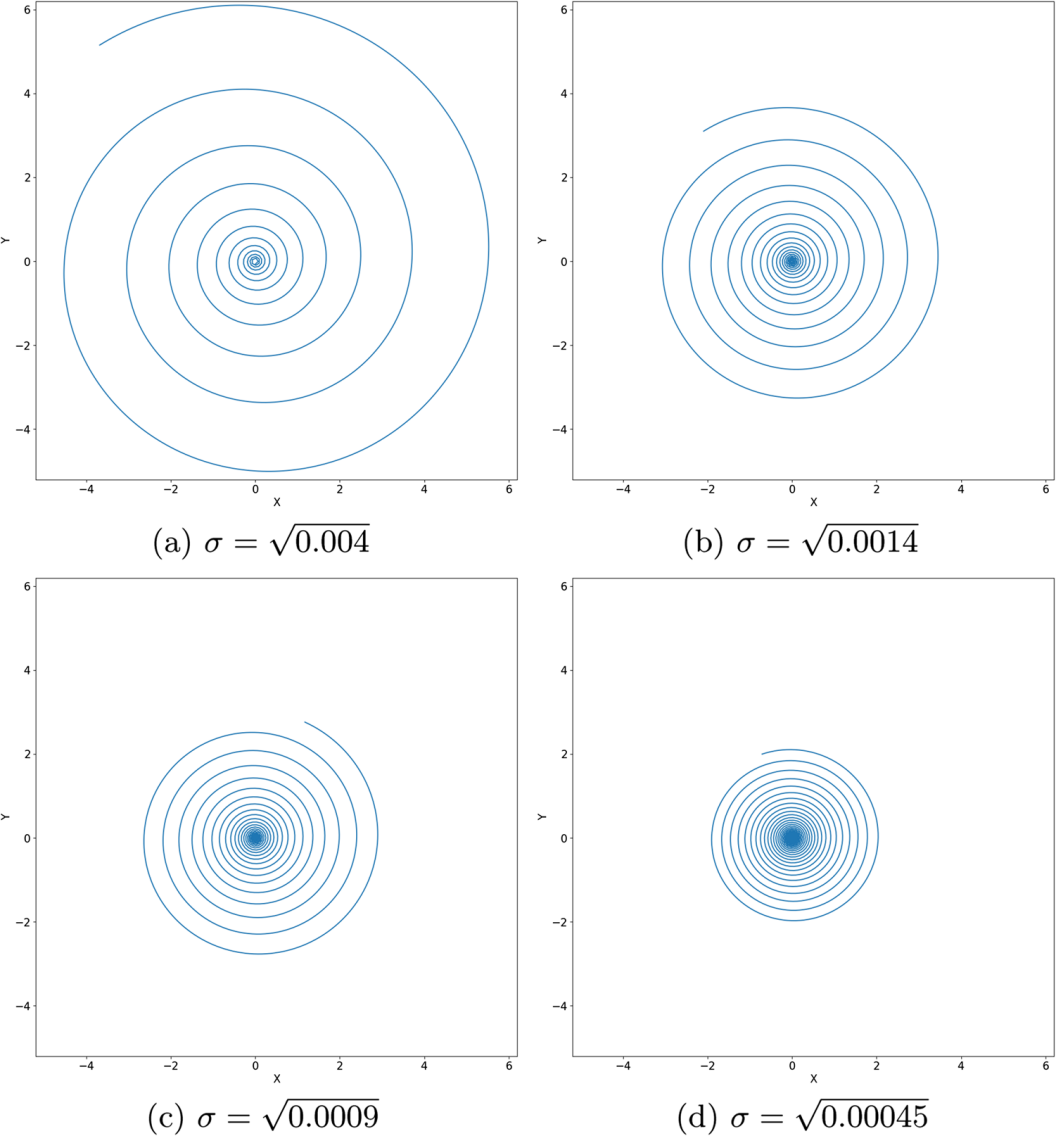
Plots of polyene curves, i.e. curves with curvature function $k(s)=\frac{1}{\sigma s}$. (a) deca-2,4,6,8-tetraene; (b) dodeca-2,4,6,8,10-pentaene; (c) tetradeca-2,4,6,8,10,12-hexaene; (d) hexadeca-2,4,6,8,10,12,14-heptaene

The polyene curves, i.e. plane curves with curvature $k(s) = \frac{1}{\sigma s}$, are depicted in Fig. 3. These spiral curves have a curvature function that resembles that used to simulate the hydrogen atom (“1D hydrogen atom as a constrained quantum dynamics problem” section). Power-law potentials $V \propto s^{-p}$ also describe interactions such as charge-charge, dipole-dipole, and also Van der Waals, a very important class of potentials in chemistry. The relationship between $\sigma$, the number of $\pi$ electrons, and the spiral geometry seems to be established, as it is evident that the curve’s pitch, i.e. the distance between two points after a revolution of the spiral, tends to decrease with the reduction of $\sigma$ and the increase in the number of $\pi$ electrons. In other words, $\sigma$ approaches zero with an increase in the number of $\pi$ electrons. This is equivalent to an increase in the geometry-induced potential, justifying the decrease in energy.

**Fig. 4:**
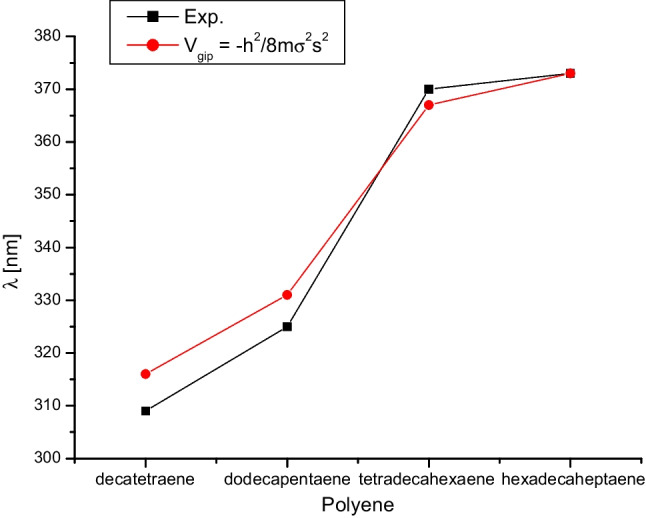
Adjustment of the parameter $\sigma$ for each polyene curve

In the case of the hydrogen atom, the direction towards the spiral’s centre indicates the nucleus’s attraction. However, for $\pi$ electrons, this association of potential to geometry is not as direct. Comparing our results with the experimental absorption values calculated using $V_{gip}$ of polyenes, we observe good agreement (Fig. 4). The errors obtained are 2.30% for deca-2,4,6,8-tetraene, 1.85% for dodeca-2,4,6,8,10-pentaene, 0.81% for tetradeca-2,4,6,8,10,12-hexaene, and 0.00% for hexadeca-2,4,6,8,10,12,14-heptaene.

Figure 4 shows that the $\pi$-$\pi ^*$ transitions are satisfactorily described with the geometry-induced potential. It is worth noting that accurate results for these transitions can also be obtained with a particle-in-a-box model using an appropriate effective mass.

## Conclusion

In this work, we employed a method to obtain the $\pi$ electron spectrum using the Jensen-Koppe-Da Costa’s confining potential formalism. Treating $\pi$ electrons as trapped in a 1D curved spiral box leads to a Schrödinger equation subjected a geometry-induced potential depending on the geometry of the corresponding spiral curve. The analytically obtained spectrum allowed us to describe the $\pi$-$\pi ^*$ transitions of the polyenes chains deca-2,4,6,8-tetraene, dodeca-2,4,6,8,10-pentaene, tetradeca-2,4,6,8,10,12-hexaene, and hexadeca-2,4,6,8,10,12,14-heptaene. The solutions are given by Bessel functions, which provide descriptions beyond the particle-in-a-box model. Studying different systems with $\pi$ resonances should demonstrate new uses of the geometry-induced potential.

## Data Availability

No datasets were generated or analysed during the current study.
